# Red Beetroot and Banana Peels as Value-Added Ingredients: Assessment of Biological Activity and Preparation of Functional Edible Films

**DOI:** 10.3390/polym14214724

**Published:** 2022-11-04

**Authors:** Danijela Šeremet, Ksenija Durgo, Anamaria Komljenović, Mihaela Antolić, Ana Mandura Jarić, Ana Huđek Turković, Draženka Komes, Božidar Šantek

**Affiliations:** Faculty of Food Technology and Biotechnology, University of Zagreb, Pierottijeva 6, 10 000 Zagreb, Croatia

**Keywords:** banana peel, red beetroot peel, biological activity, edible films

## Abstract

In the present study, water extracts from banana and red beetroot peels were evaluated as a potential source of biologically active compounds for the formulation of edible films. Using spectrophotometric and HPLC-DAD methodologies, banana peel extract was found to be a valuable source of dopamine (156.08 mg L^−1^), while red beetroot peel extract was abundant in red-violet pigments betacyanins (90.1 mg betanin L^−1^). The biological activity of the extracts was studied by determining their effects on macromolecular models, including DNA (plasmid phiX RF1 DNA), protein (bovine serum albumin), and lipid (linoleic acid) models, as well as on continuous human cell lines of colon cancer Caco-2 and hepatocellular liver cancer Hep G2 at concentrations of 0.2 and 1 mg mL^−1^. Results showed that the extracts had no adverse effects and both were further used for the formulation of edible films using alginate in combination with three types of plant proteins—rice, peanut, and pumpkin. In general, edible films based on banana peel extract were characterized by better bioactive properties compared with the films based on red beetroot peel extract. The addition of peanut proteins into the formulations resulted in the most desirable bioactive profile of the formulated edible films, including total phenolic content and antioxidant capacity. Aside from the control sample prepared only with the alginate, the highest dopamine content was determined in the film with incorporated pumpkin proteins (10.72 mg g^−1^ dw), while the sample prepared with peanut proteins was richest in betacyanins (175.58 mg betanin g^−1^ dw).

## 1. Introduction

Edible films can be defined as layers formed separately as thin sheets and wrapped over the food surface. The major advantage of edible films is that they are made from ingredients that are safe for human consumption and therefore can be eaten along with the food products while helping to reduce the problem of waste disposal [1]. Hydrocolloids, including polysaccharides and proteins, are the most commonly used biopolymers in the production of edible materials, whether they are of animal, plant or microbial origin, and improvement of their water vapor barrier properties can be achieved by incorporating various types of oils and fats [2]. Colorant, flavors, anti-browning agents, nutrients, spices, and antimicrobial and antioxidant compounds can be added as active compounds in the formulation of edible films. When edible films deliver or absorb these compounds into or from the packaged food to extend shelf-life or improve the condition of the packaged food, they can be considered as active food packaging. Recently, interest has shifted to compounds of natural origin as some of the synthetic antioxidants have been associated with adverse health effects. Many studies are being conducted on the use of extracts from agro-industrial wastes, such as fruit and vegetable residues, as a source of bioactive compounds for the production of active edible packaging, thus contributing to the circular economy and bioeconomy [3]. It is estimated that nearly one billion metric tons of food waste was produced globally in 2019 [4]. In the food industry, waste is generated during the processing of vegetable and animal raw materials into foodstuffs, which generally consists of extracting or separating the nutritious part from the residues with low nutritional value or inedible components [5]. A large proportion of food waste is also generated in households—in 2019, as much as 61% of food waste came from households [4]. Agro-industrial waste can serve as a natural material for the extraction of different bioactive compounds, such as pigments, vitamins, phenolic compounds, etc., since the outer layers of fruits and vegetables, including husks, peels, and shells, often have a higher content of these compounds than the inner parts [6,7]. Namely, most of these bioactive compounds play a preventive role in various diseases associated with oxidative stress and they also possess strong antioxidant properties [8].

Fruits and vegetables accounted for 38% of the volume of food losses and waste globally in 2017 [9], pointing to the need for new recycling options to help reduce food waste. Banana (*Musaceae*) is one of the most consumed fruits in the world [10] with an annual production of 119 million metric tons in 2020 [11]. About 30% of banana fruit consist of inedible peels [12] and it is obvious that a lot of peel waste is generated annually in the fruit industry and households worldwide. Banana peel is a rich source of phenolic compounds, especially flavonols, flavan-3-ols, and catecholamines [13]. Red beetroot (*Beta vulgaris*) is one of the vegetables with the highest antioxidant activity, mainly due to the nitrogenous red-purple pigments, betacyanins and betanin in particular. Since the use of betanin as a food colorant was approved by EFSA and FDA [14] and due to the increasing demand for natural food colorants, the worldwide consumption of beetroot extracts has increased [15]. The amount of betanin in beetroot peel is higher than in the flesh and crown [16], indicating a possible reuse of red beetroot peels.

In the European Union, active edible packaging is regulated by several regulations and even by some related to food additives. Active compounds added to food packaging are considered indirect food additives as they are not added directly to the food [3]. Therefore, all active compounds should be tested for their potential adverse health effects before final application and incorporation into edible films. In the present study, banana and red beetroot peels were evaluated as potential materials for the enrichment of edible films with antioxidants of natural origin. For that purpose, water extracts of banana and red beetroot peels were firstly subjected to the analysis of biological activity through examining the effects on genetic material using a DNA model (plasmid phiX RF1 DNA) and continuous human cell lines of colon cancer Caco-2 and hepatocellular liver cancer Hep G2 as test systems, as well as protein (bovine serum albumin) and lipid (linoleic acid) models. Finally, formulated edible films were investigated through characterization of phenolic composition and determination of physical and mechanical parameters.

## 2. Materials and Methods

### 2.1. Materials and Chemicals

Banana (var. Cavendish; Costanza organic bananas, Ecuador) and red beetroot were purchased in a local store. Rice (fat content: 4.5%, carbohydrates content: 2.9%, and protein content: 83.0%), peanut (fat content: 13.3%, carbohydrates content: 26.6%, and protein content: 53.3%), and pumpkin (fat content: 10.8%, carbohydrates content: 17.3%, and protein content: 51.4%) protein powders were obtained from Nutrigold (Zagreb, Croatia).

Folin–Ciocalteu’s reagent, sodium chloride, sodium hydroxide, dimethyl sulfoxide, and hydrogen peroxide were supplied from Kemika (Zagreb, Croatia). Dopamine hydrochloride, (S)-6-Methoxy-2,5,7,8-tetramethylchromane-2-carboxylic acid (Trolox), 2,2-Diphenyl-1-picrylhydrazyl (DPPH), 2,20-Azino-bis(3-ethylbenzothiazoline-6-sulfonic acid) diammonium salt (ABTS), gallic acid (>97%), alginic sodium salt from brown algae (sodium alginate, low viscosity), pepsin from porcine gastric mucosa, bile bovine, pancreatin from porcine pancreas, low melting point agarose, normal melting point agarose, EDTA disodium salt (Na_2_EDTA), N-lauroylsarcosine sodium salt, linoleic acid, trichloroacetic acid, guanidine hydrochloride, 2,4-dinitrophenylhydrazine (DNPH), bovine serum albumin, Triton X-100, and ethidium bromide were purchased from Sigma-Aldrich (St. Louis, MO, USA). Methanol was supplied from Panreac (Barcelona, Spain), while ethanol, formic acid, and acetonitrile from Carlo Erba (Val de Reuil, France). Potassium dihydrogen phosphate, sodium hydrogen carbonate, magnesium chloride hexahydrate, ammonium carbonate, and ethyl acetate were purchased from Lach-ner (Neratovice, Czech Republic). Ferrous sulphate and ammonium thiocyanate were supplied by Gram-mol d.o.o. (Zagreb, Croatia) and ascorbic acid from T.T.T. d.o.o. (Sveta Nedelja, Croatia). F12 medium, bovine serum, and fetal bovine serum were purchased from Capricorn Scientific GmbH (Hessen, Germany.) Plasmid Phi174, RF DNA was purchased from Promega (Fitchburg, WI, USA). All chemicals used for experimental procedures were of analytical or HPLC grade.

### 2.2. Methods

#### 2.2.1. Preparation of Plant Materials

The stage of ripeness of bananas was determined using the standard color chart of Tapre and Jain [17] and scored a value of 6 (full yellow). Bananas were peeled and the peels were immersed in boiling water and blanched for 7 min. The peels were then dried with paper towels and freeze-dried (Alpha 1-2 LD plus freeze-dryer, Martin Christ, Germany). Red beetroots were peeled, and the peels were air-dried at room temperature for 48 h. The dried banana and red beetroot peels were ground and sieved to obtain fractions <450 µm for experiments and analyses.

#### 2.2.2. Preparation of Extracts of Plant Materials

Extracts of banana and red beetroot peels were prepared in a water bath (Inko VKZ ERN, Inkolab d.o.o., Zagreb, Croatia) for 10 min at 100 °C using a sample/solvent ratio 1g/50 mL. Distilled water was used as a solvent. After the extraction, centrifugation at 9500 rpm for 20 min (Thermo Scientific SL8/8R centrifuge, Waltham, MA, USA) was performed and supernatants were collected and immediately subjected to analysis.

#### 2.2.3. Bioactive Characterization of Extracts of Plant Materials

Obtained extracts were subjected to spectrophotometric (Genesys 10S UV-VIS Spectrophotometer, Thermo Fisher Scientific, Waltham, MA, USA) analyses of total phenolic content (TPC) [18], DPPH radical scavenging activity [19], and ABTS^•+^ decolorization assay [20]. A detailed description of these methods can be found elsewhere [21].

Identification and quantification of dopamine in banana peel extract was performed on an Agilent Series 1200 chromatographic system (Agilent Technologies, Santa Clara, CA, USA) with a Zorbax Extend C18 chromatographic column (4.6 mm, 250 mm, 5 m i.d.) (Agilent Technologies, Santa Clara, CA, USA) coupled with a Photodiode Array Detector (PAD) (Agilent Technologies, Santa Clara, CA, USA) following the method described by Šeremet et al. [21]. The chromatograms were recorded at 280 nm. The analysis for all samples was performed in duplicate. All samples were filtered through a 0.45 µm membrane filter (Nylon Membranes, Supelco, Bellefonte, PA, USA) prior to the analysis. Identification of dopamine was performed by comparing the retention time and characteristic absorption spectrum (190–400 nm) with commercially available standards, while quantification was enabled by establishing calibration curves (2–100 µg mL^−1^).

The total betacyanin content in the obtained red beetroot peel extract was determined spectrophotometrically (Genesys 10S UV-VIS Spectrophotometer, Thermo Fisher Scientific, Waltham, MA, USA) by measuring absorption values at 538, as described previously [22]. The results were expressed as equivalents of betanin.

#### 2.2.4. Preparation of Extracts for Biological Analyses

The prepared extracts of banana and red beetroot peels were evaluated for biological activity using macromolecular models and human cell lines. In addition, extracts with removed polysaccharides were also prepared and analyzed since polysaccharides can have different roles in bioavailability of polyphenols as they can modulate cellular processes or can be bound to other bioactive compounds and change their bioavailability [23]. Polysaccharides in the extracts were removed by ethanol precipitation (4-fold volume of 96% ethanol). The precipitation lasted 1 h, and the precipitated polysaccharides were removed by filtration (Whatman^®^ filter papers 4). Both extracts, with and without polysaccharides, were evaporated under vacuum (Buchi Rotavapor R124, Flawil, Switzerland) and freeze-dried (Christ, Alpha 1-2 LD plus, Osterode, Germany). Working solutions were prepared at concentrations of 0.2 and 1 mg mL^−1^.

#### 2.2.5. Biological Analyses of Extracts

##### Biological Test Systems

The double-stranded plasmid phiX RF1 DNA, with a length of 5,386 base pairs and a molecular weight of 3.5 × 106 Da, isolated from *E. coli* [24] was used to test the antioxidant effect of banana and red beetroot peel extracts on DNA against hydroxyl radicals generated by hydrogen peroxide and UV light. Adenocarcinoma colon cells (CaCo-2) and hepatocellular carcinoma cells (HepG2) (kindly provided by the Institute for Medicinal Research, Zagreb, Croatia) were used to determine the genotoxic effect of banana and red beetroot peel extracts. Cells were grown as monolayer cultures in F-12 medium supplemented with 10% (*w*/*v*) fetal bovine serum, 4500 mg L^−1^ glucose, and 1% (*w*/*v*) penicillin/streptomycin [25].

##### Effect of Extracts on DNA Model: Plasmid phiX174 RF1 DNA

A 1% (*w*/*v*) agarose gel was prepared in a TAE buffer. The agarose and buffer were melted to boiling point in a microwave oven and cooled to 60 °C. The homogeneous agarose was transferred to the mold and a comb was immediately used to form the wells. Samples for electrophoresis contained 10 μg mL^−1^ of plasmid, 0.1% H_2_O_2_, and extracts of banana and red beetroot peel in final concentrations of 0.2 and 1 mg mL^−1^. The final volume was set with a TAE buffer to 30 μL. Samples were irradiated with UV radiation for 3 min at 2 J m^−2^ s^−1^. A negative control contained 10 μg mL^−1^ of plasmid. A positive control contained 10 μg mL^−1^ of plasmid, 0.1% H_2_O_2_, and it was irradiated for 3 min at 2 J m^−2^ s^−1^. After irradiation, samples for electrophoresis were prepared by adding the loading buffer. Electrophoresis was run for 60 min at 110 mA. After electrophoresis, the gel was stained with ethidium bromide solution at a concentration of 20 μL mL^−1^ for 15 min, irradiated with UV radiation for 10 min, and visualized under UV light.

##### Alkaline Comet Assay

The comet assay was performed under alkaline conditions as described elsewhere [26] with some modifications. Two replicate slides were prepared for each sample. Agarose gels were prepared on fully frosted slides coated with 1% (*w*/*v*) and 0.6% (*w*/*v*) normal melting point (NMP) agarose. The adenocarcinoma colon cell line and hepatocellular carcinoma cells were seeded in Petri dishes at a concentration of 10^5^ cells mL^−1^. Cells were treated with banana and red beetroot peel extracts with and without polysaccharides for 24 h. After incubation, medium containing extracts was expelled, the cells were washed 3 times with cold phosphate-buffered saline, and the pellet was collected. A measure of 10 μL of the cell suspension was mixed with 0.5% (*w*/*v*) low melting point (LMP) agarose, placed on slides and covered with a layer of 0.5% (*w*/*v*) LMP agarose. The slides were immersed for 1 h in freshly prepared ice-cold lysis solution (2.5 M NaCl, 100 mM Na_2_EDTA, 10 mM Tris-HCl, 1% Na-sarcosinate, pH 10) with 1% Triton X-100 and 10% dimethyl sulfoxide. Alkaline denaturation and electrophoresis were performed at 4 °C in a freshly prepared electrophoresis buffer (300 mM NaOH, 1 mM Na_2_EDTA, pH 13.0). After 20 min of denaturation, slides were randomly placed side by side in the horizontal gel electrophoresis tank facing the anode. Electrophoresis at 25 V lasted another 20 min. After electrophoresis, the slides were washed with a neutralization buffer (0.4 M Tris-HCl, pH 7.5) 3 times at 5-min intervals. Slides were stained with ethidium bromide (20 μg mL^−1^) and stored at 4 °C in humidified sealed containers until analysis. Each slide was examined using a 250× magnification fluorescence microscope (Zeiss) equipped with an excitation filter of 515–560 nm and a barrier filter of 590 nm. The microscope image was transferred to a computer-based image analysis system (Comet Assay II, Perceptive Instruments Ltd.). The comet parameter analyzed was tail intensity (DNA %). A total of 100 comets per sample were scored.

##### Effect of Extracts on Protein Model: Bovine Serum Albumin

The degree of protein oxidation was measured as formation of 2,4-dinitrophenylhydrazone from the reaction of 2,4-dinitrophenylhydrazine (DNPH) with carbonyl groups of proteins. The resulting 2,4-dinitrophenylhydrazone contains a dinitrophenyl group (DNP), which is detected spectrophotometrically at 370 nm [27]. The reaction mixtures contained banana and red beetroot peel extracts with and without polysaccharides in concentrations of 0.2 and 1 mg mL^−1^, 60 μL BSA (bovine serum albumin) at a concentration of 40 mg mL^−1^, 60 μL FeCl_3_ at a concentration of 0.5 mM, 60 μL of ascorbic acid, 60 μL H_2_O_2_ concentration of 10 mM, and distilled water to a total volume of 600 μL of reaction mixture. The control contained 60 μL methanol. All samples were prepared in triplicate and incubated for 30 min at 37 °C. After incubation, 500 μL DNPH was added and incubated at room temperature for 15 min. Then 500 μL of 10% trichloroacetic acid was added and incubated on ice for 15 min. After incubation, the supernatant was discarded, and the precipitate was washed with 1 mL of ethyl acetate. All samples were resuspended in 1 mL of 6 M guanidine-HCl, and the absorbance was measured at 370 nm. All samples were prepared in 3 parallels and the experiment was repeated 3 times.

##### Effect of Extracts on Lipid Model: Linoleic Acid

The reaction mixture contained a linoleic acid emulsion at a concentration of 0.3 mg mL^−1^ and banana and red beetroot peel extracts with and without polysaccharides at concentrations of 0.2 and 1 mg mL^−1^. A sodium phosphate buffer was used to adjust the final volume. The positive control contained 125 μL methanol in place of the extract. The tested samples were incubated at 37 °C for 50 h. After incubation, 2 mL ethanol (75%), 50 μL ammonium thiocyanate (30%, *w*/*v*), and 50 μL ferrous sulphate (0.02 M, diluted in 3.5% HCl) were added to 50 μL of the reaction mixture. After 3 min, the absorbance was measured at 500 nm. All samples were prepared in 3 parallel experiments and the experiment was repeated 3 times.

#### 2.2.6. Preparation of Edible Films: Casting Technique

Ingredients used for the preparation of edible films are presented in Table 1. In the case of control films, ingredients were prepared in distilled water (W), while in others, water extracts (E) of banana and red beetroot peel were used instead (prepared as described in Section 2.2.2.). Mixed ingredients were left on a magnetic stirrer at an ambient temperature overnight until complete dissolution. All film solutions, 10 g each, were cast onto a Petri dish (diameter = 10 cm) and allowed to dry at room temperature for at least 3 days.

#### 2.2.7. Bioactive Characterization of Edible Films

A known amount of prepared edible films was dissolved in distilled water. A solution was then added in 4-fold to a volume of 96% ethanol to precipitate the biopolymers. After centrifugation (Thermo Scientific SL8/8R centrifuge, Waltham, MA, USA) at 9500 rpm for 10 min, clear supernatants were used for bioactive characterization of edible films performing the methods described in Section 2.2.3. Simulative gastro-intestinal digestion of formulated edible films was performed as described elsewhere [21].

#### 2.2.8. Physical and Mechanical Characterization of Edible Films

The soluble dry matter of the edible films was determined by dissolving a known amount of the formulated film in distilled water and measuring it on a digital refractometer Abbemat 3000 (Anton Paar, Graz, Austria). The thickness of the films was measured using a digital handheld micrometer Micromar 40 EX (Mahr GmbH, Göttingen, Germany) on 6 random locations on each film. The analysis of the mechanical properties was performed using a TA.HDplus texture analyzer (Stable Micro Systems, Godalming, UK) on the films cut into 10-mm-wide and 2-mm-long strips. Color determination was performed using a portable spectrophotometer CM-700d (Konica Minolta, Japan). Results were expressed as CIE coordinates of L* (lightness), a* (redness/greenness), and b* (yellowness/blueness). Overall color difference (Δ*E*) was calculated from the ΔL*, Δa*, and Δb* using sample C_A as the control (Equation (1)):

(1) $$\Delta E=\sqrt{\Delta L^{*2}+\Delta a^{*2}+\Delta b^{*2}}$$

The color deviation in comparison with the control sample (sample C_A) was rated according to the following range: Δ*E* < 0.2 (no visible color difference), Δ*E* = 0.2–1.0 (noticeable color difference), Δ*E* = 1–3 (visible color difference), Δ*E* = 3–6 (well visible color difference), and Δ*E* > 6 (apparent color deviation) [28].

#### 2.2.9. Statistical Analysis

All results are presented as mean +/− standard deviation, except for genotoxicity assays (comet assay), where results are presented as box and whisker plots. X represents the mean of the tail intensity, the lower line is the minimum, the middle line represents the median tail intensity, and the area above the median is the third quartile of the tail intensity data. The upper whisker is the maximum value measured by the comet assay.

Statistical analysis of the results obtained in the experiments in Section 2.2.5 was performed using the programme JASP 16.0 (Jasp, Amsterdam, The Netherlands). One-way ANOVA and the Sheffe post-hoc test with a significance level of α = 0.05 were applied. Results from the experiments in Section 2.2.7 and Section 2.2.8 were analyzed using SPSS Statistics 17.0 software (IBM, Armonk, New York, NY, USA) with a significance level of α = 0.05 using one-way ANOVA and Tukey’s post-hoc test.

## 3. Results and Discussion

### 3.1. Bioactive Characterization of Banana and Red Beetroot Peel Extracts

The extracts obtained from banana and red beetroot peels were analyzed for total phenolic content (TPC) and antioxidant capacity. The extract of banana peel was additionally analyzed for dopamine content, while the extract of red beetroot peel was subjected to the analysis of total betacyanin content. The results are presented in Table 2.

Both extracts were found to be a rich source of phenolic compounds, although the highest content of TPC with a value of 507.1 mg GAE L^−1^ was detected in the banana peel extract, compared to the red beetroot peel extract with a value of 239.6 mg GAE L^−1^. The prominent antioxidant properties of the extracts were also evidenced by the good scavenging activity against ABTS (2.34 and 1.69 mmol TroloxE L^−1^, respectively) and DPPH (1.52 and 1.04 mmol TroloxE L^−1^, respectively) radicals. Dopamine is an antioxidant characteristic for banana peel, and belongs to the group of catecholamines, whose antioxidant activity is related to their *o*-dihydroxy structure, while amino residue favors their hydrophilic character [12]. In the present study, the dopamine content in banana peel extract was 156.8 mg L^−1^. A high content of dopamine in banana peels was also reported by Kanazawa and Sakakibara [29] with a range of 80–560 mg per 100 g and by González-Montelongo et al. [12] with up to 341 mg per 100 g dw depending on the banana cultivars and extraction conditions. Further, red beetroot is characterized by nitrogenous red-purple color pigments—betacyanins with betanin being the most abundant [30]. In the present study, red beetroot peel also proved to be a valuable source of these compounds, since its extract contained 90.1 mg betanin L^−1^. A high content of betacyanins in red beetroot peel was previously reported by Sawicki et al. [31] with 12.79 mg betanin g^−1^ dw.

### 3.2. Biological Activity of Banana and Red Beetroot Peel Extracts

The prepared extracts of banana and red beetroot peels were further evaluated for their biological activity by testing the protective effects on genetic material using a DNA model (plasmid phiX RF1 DNA) and continuous human cell lines of colon cancer Caco-2 and hepatocellular liver cancer Hep G2, as well as protein (BSA) and lipid (linoleic acid) models.

**Figure 1:**
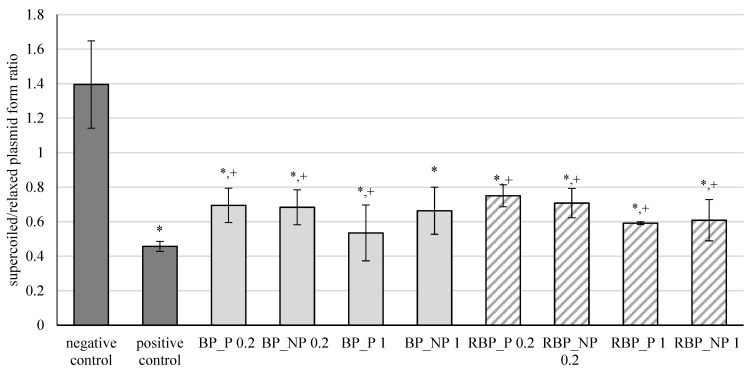
Effect of banana and red beetroot peel extracts against hydroxyl radicals generated with UV and hydrogen peroxide on DNA model. Results are presented as the mean value +/− standard deviation of 3 independent experiments. BP_P-banana peel extract with polysaccharides; BP_NP-banana peel extract without polysaccharides; RBP_P-red beetroot peel extract with polysaccharides; RBP NP-red beetroot peel extract without polysaccharides, concentrations 0.2 and 1 mg mL^−1^; *—statistically significant (*p* < 0.05) difference in comparison to negative control; +—statistically significant (*p* < 0.05) difference in comparison to positive control.

The results of the effects of banana and red beetroot peel extracts, with (BP_P and RBP_P, respectively) and without (BP_NP and RBP_NP, respectively) polysaccharides, on oxidative damage of the DNA model caused by hydroxyl radicals are shown in Figure 1. The results are expressed as the ratio between supercoiled and relaxed plasmid forms.

Oxidation of DNA molecules is a consequence of the activity of the generated reactive oxygen compounds. Furthermore, different xenobiotics can act as oxidative agents once they are metabolically activated. Cells have efficient DNA repair mechanisms, but damage caused by oxidation remains during replication and can ultimately cause mutations [32]. From the presented results, it is evident that banana and red beetroot peel extracts did not prevent a statistically significant inhibition of the oxidative damage of the DNA model. Nevertheless, it can be concluded that some protection of the supercoiled plasmid was maintained, as these levels are higher than the positive control. Furthermore, it can be concluded that the presence of polysaccharides in the extracts did not play a role in protecting the genetic material, as all concentrations tested (0.2 and 1 mg mL^−1^) protected the DNA from attack by hydroxyl radicals to a similar extent. Nevertheless, the samples studied prevented unwinding of supercoiled DNA to some extent.

**Figure 2:**
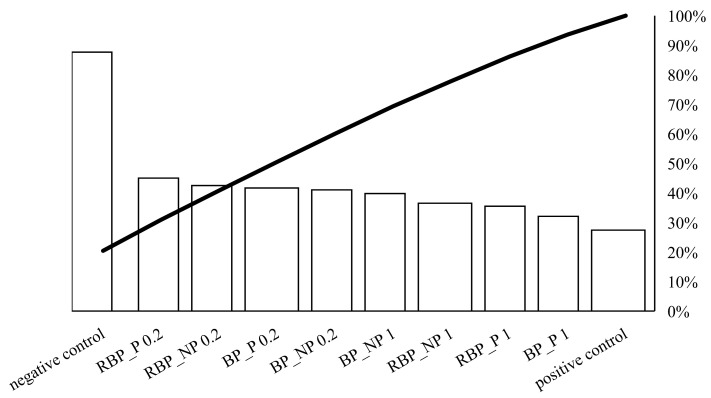
Pareto chart showing the trend of the protective effect of banana and red beetroot peel extract against hydroxyl radicals generated with UV and hydrogen peroxide on a DNA model. BP_P-banana peel extract with polysaccharides; BP_NP-banana peel extract without polysaccharides; RBP_P-red beetroot peel extract with polysaccharides; RBP NP-red beetroot peel extract without polysaccharides, concentrations 0.2 and 1 mg mL^−1^.

On Figure 2, the individual values of the potential protective effect of the extracts are represented by bars in descending order, while the total cumulative effect of the sample is represented by a curved line.

Crucial for the protective effect was the concentration of 0.2 mg mL^−1^. The presence of polysaccharides did not play a decisive role in the protective effect. Higher concentrations (1 mg mL^−1^) showed a lower protective effect against DNA unwinding, but it is worth noting that both banana and red beetroot peel extracts without polysaccharides (BP_NP and RBP_NP) had a stronger effect against hydroxyl radicals than the samples with polysaccharides (BP_P and RBP_P). The significance of these results lies in the fact that the DNA was not damaged more than in the positive control and all the samples studied protected the DNA from linearization.

Furthermore, the genotoxic effect of banana and red beetroot peel extracts was investigated. For this purpose, two metabolically active human cell lines were used as biological test systems—human adenocarcinoma cell line CaCo2 and hepatocellular carcinoma cell line HepG2. After treatment with the investigated extract, a comet assay was performed, and the tail intensity was a parameter for the degree of DNA damage. The obtained results were compared with the negative control.

**Figure 3:**
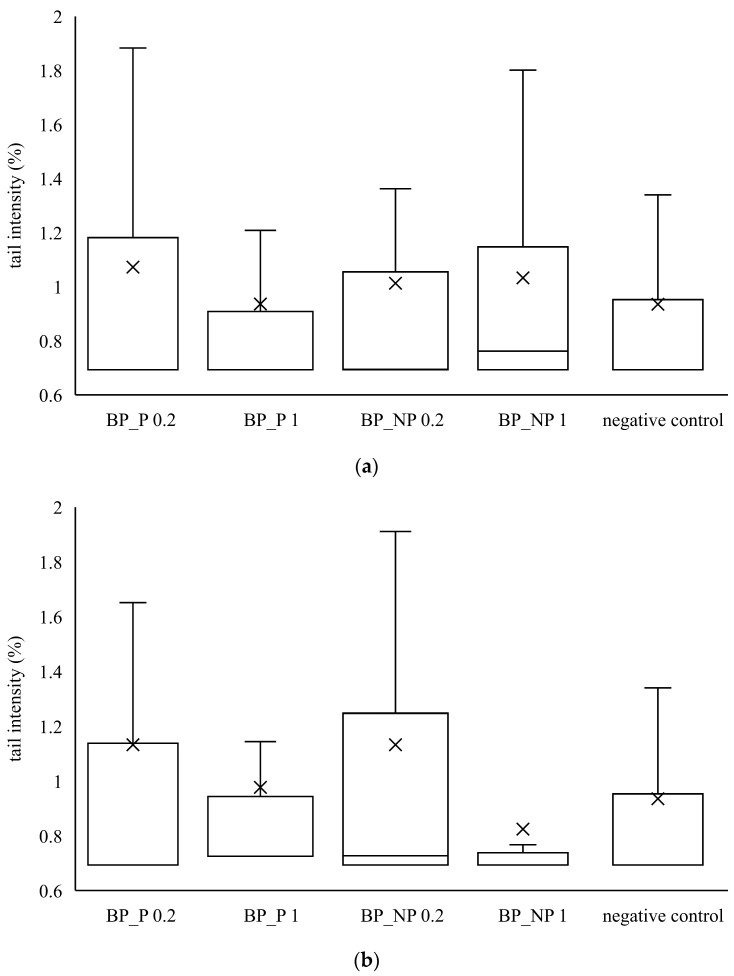
Genotoxic effect of banana peel extract on (**a**) CaCo2 cells and (**b**) HepG2 cells. A box and whisker plot summary of a set of tail intensity data obtained in 100 comets. X represents the mean value of tail intensity, the bottom line is the minimum, the middle line represents the median value of tail intensity, and the area above the median line is the third quartile of tail intensity data. The upper whisker is the maximum value measured by the comet assay. BP_P-banana peel extract with polysaccharides; BP_NP-banana peel extract without polysaccharides, concentrations 0.2 and 1 mg mL^−1^.

It can be seen from Figure 3 that none of the samples of banana peel extract caused a statistically significant increase in tail intensity in HepG2 and CaCo2 cells. The distribution of the data is not symmetrical because the median is not in the middle of the boxes and the whiskers on both sides of the box are not equal. The median value is closer to the bottom of the box, so it can be concluded that the distribution is positively skewed. It means that the mean value is higher than the median one, indicating that tail intensity data measured in all samples is more towards the lower side and the mean average of all the values.

**Figure 4:**
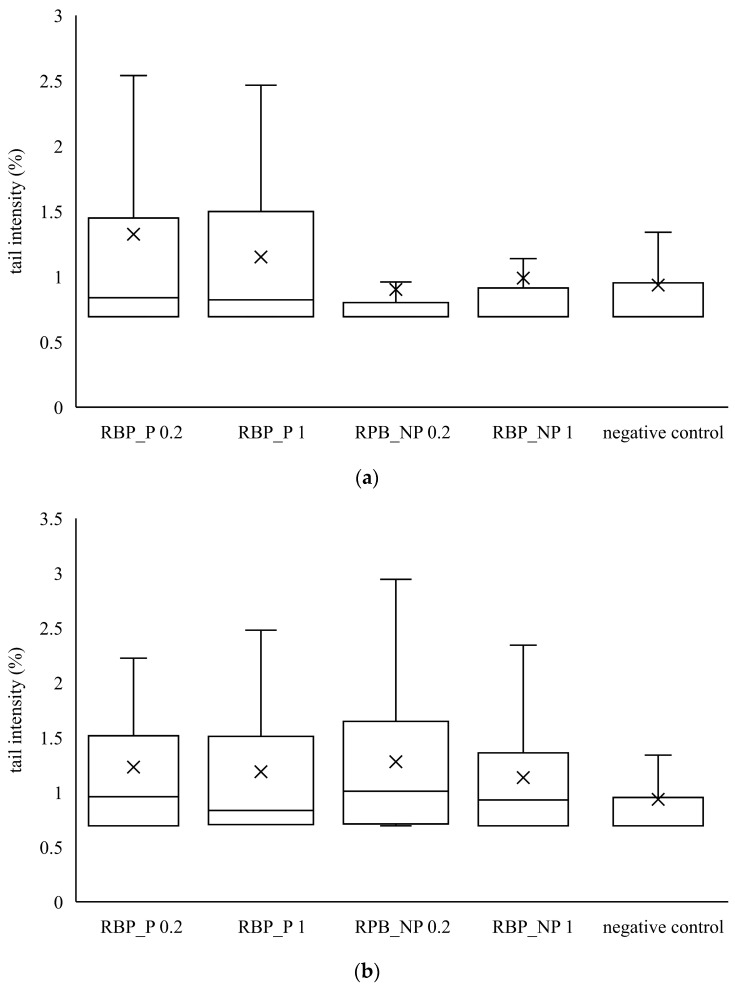
Genotoxic effect of red beetroot peel extract on (**a**) CaCo2 cells and (**b**) HepG2 cells. A box and whisker plot summary of a set of tail intensity data obtained in 100 comets. X represents the mean value of tail intensity, the bottom line is the minimum, the middle line represents the median value of tail intensity, and the area above the median line is the third quartile of the tail intensity data. The upper whisker is the maximum value measured by the comet assay. RBP_P-red beetroot peel extract with polysaccharides; RBP_NP-red beetroot peel extract without polysaccharides, concentrations 0.2 and 1 mg mL^−1^.

Similar to banana peel extracts, red beetroot peel extracts did not cause a statistically significant increase in tail intensity compared to the control. From the data in Figure 3 and Figure 4, it is evident that the banana and red beetroot peel extracts have no genotoxic effect. The presence of polysaccharides did not play a role in the potential genotoxicity of the samples, so it can be assumed that these extracts can be used safely in investigated concentrations. Edziri et al. [33] investigated the methanolic and juice extract of red beetroot for their antioxidant, anticoagulant, and genotoxic activities. The extract showed antioxidant activities but there was no genotoxic effect monitored.

**Figure 5:**
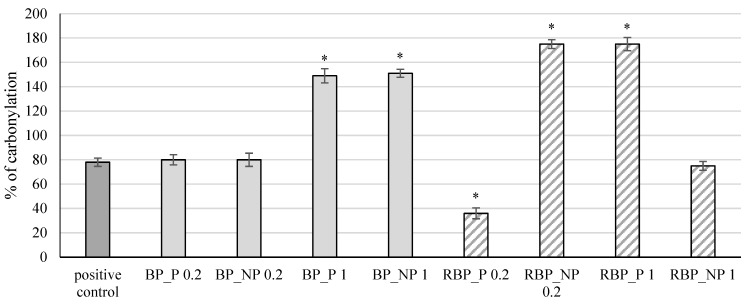
Effect of banana and red beetroot peel extracts on protein carbonylation. Results are presented as the mean value +/− standard deviation of 3 independent experiments. BP_P-banana peel extract with polysaccharides; BP_NP-banana peel extract without polysaccharides; RBP_P-red beetroot peel extract with polysaccharides; RBP_NP-red beetroot peel extract without polysaccharides, concentrations 0.2 and 1 mg mL^−1^; *—statistically significant (*p* < 0.05) difference in comparison to positive control.

The extracts were further tested for their effect on protein carbonylation, and the results are shown in Figure 5.

Protein side chains, especially those containing tryptophan, histidine, and cysteine, are particularly sensitive to fragmentation caused by the action of free radicals which can lead to the inactivation of enzymes and receptors resulting in irreversible cellular damage [34,35]. The presence of carbonyl groups represents the first step in the oxidation of proteins, and the method of measuring the formed carbonyl groups is often used to test protein damage caused by oxidation. The banana peel extracts at a concentration of 1 mg mL^−1^, both with (BP_P) and without polysaccharides (BP_NP), showed a statistically significant (*p* < 0.05) increase in the percentage of protein carbonylation (149% and 151%, respectively) compared with the positive control (78%), whereas at lower concentrations the change was not significant (*p* > 0.05). The red beetroot peel extract with polysaccharides (RBP_P) at a concentration of 0.2 mg mL^−1^ had an antioxidant effect, decreasing the percentage of carbonylation to 36%, while the same concentration without polysaccharides (RPB_NP 0.2) and a higher concentration with polysaccharides (RPB_P 1) showed a significant (*p* < 0.05) prooxidative effect (174% and 175%, respectively) compared with the control.

**Figure 6:**
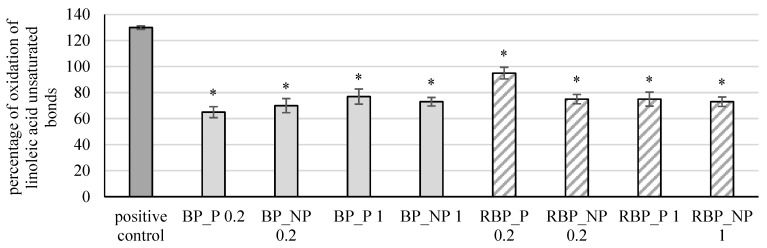
Effect of banana and red beetroot peel extracts on damage of linoleic acid. Results are presented as the mean value +/− standard deviation of 3 independent experiments. BP_P-banana peel extract with polysaccharides; BP_NP-banana peel extract without polysaccharides; RBP_P-red beetroot peel extract with polysaccharides; RBP NP-red beetroot peel extract without polysaccharides, concentrations 0.2 and 1 mg mL^−1^; *—statistically significant (*p* < 0.05) difference in comparison to positive control.

The extracts were further tested for their effect on the protective effect on linoleic acid damage, and the results are shown in Figure 6.

Banana and red beetroot peel extracts with and without polysaccharides in all tested concentrations reduced significantly (*p* < 0.05) the percentage of linoleic acid peroxidation. In the case of banana peel extract, the highest protective effect was noted in the sample with polysaccharides at a concentration of 0.2 mg mL^−1^ (BP_P 0.2; 65%), while for red beetroot peel extracts, it was the sample without polysaccharides a concentration of 1 mg mL^−1^ (RBP_NP 1; 73%). Zhu et al. [36] investigated changes in a bovine serum albumin (BSA) protein model incubated with polyunsaturated fatty acids (PUFA) and examined the influence of polyphenols (including flavonoids and carotenoids) on PUFA. They concluded that polyphenols are effective in slowing lipid peroxidation and that they can prevent the effect of free radicals and lipid peroxidation-induced alteration of BSA.

### 3.3. Physical and Mechanical Characterization of Edible Films

**Figure 7:**
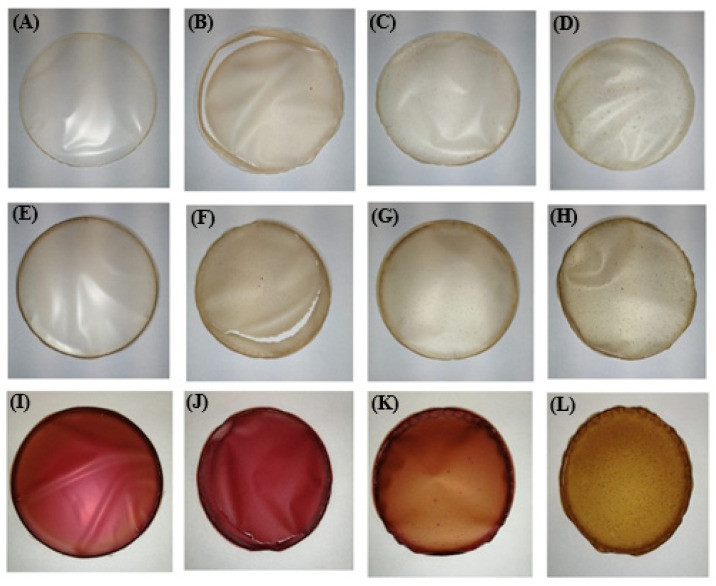
Visual appearance of formulated edible films: (**A**): C_A, (**B**): C_RIC, (**C**): C_PEA, (**D**): C_PUM, (**E**): BP_A, (**F**): BP_RIC, (**G**): BP_PEA, (**H**): BP_PUM, (**I**): RBP_A, (**J**): RBP_RIC, (**K**): RBP_PEA, (**L**): RBP_PUM.

The visual appearance of the edible films is shown in Figure 7. All films were easily removed from the Petri dishes, but some cracking occurred in the films prepared with rice proteins (samples C_RIC, BP_RIC and RBP_RIC).

Physical characterization of the edible films included determination of soluble dry matter, thickness, and color parameters, while mechanical parameters included firmness and tensile strength. The results are shown in Table 3.

The soluble dry matter ranged from 67.0% in sample RPB_RP to 95.2% in sample RBP_PP, while thickness was in the range of 40.2 µm (sample RBP_PP)—153.3 µm (sample C_PB). It is worth noting that the thinnest films were those with pumpkin proteins, regardless of whether they were prepared in water (56.7 µm), banana peel (63.7 µm), or red beetroot peel (40.2 µm) extract, and the thickest were those with peanut proteins (153.3, 138.9, and 124.2 µm, respectively). Similar results regarding the thickness of edible films were reported by Gómez-Estaca et al. [37], who measured 100 and 105 µm for sole and catfish gelatine films, respectively. Considering the color parameters of the control samples, it is obvious that incorporation of plant proteins resulted in a significant decrease (*p* < 0.05) in parameter L*, compared with the samples prepared only with alginate (sample C_A), which means that they were darker and consistent with their visual appearance (Figure 7). The addition of banana peel extract (BP) led to a further decrease in parameter L*, while the darkest films with the lowest parameter L* were the ones prepared with the red beetroot peel extract (RPP). The positive values of parameter a* correspond with the red color, and the high values of this parameter were found in the samples prepared with red beetroot peel extract (RBP), which is consistent with their visual appearance (Figure 7). The apparent color deviation (Δ*E* > 6) was observed in all samples and was most pronounced in the films prepared with the red beetroot peel extract (RBP).

Proper mechanical properties are the basic requirements for edible films, as low flexibility or strength can result in premature failure or cracking during manufacturing, handling, or use [38]. The highest value of firmness was observed in the control sample (C_PP) prepared with pumpkin proteins (31.0 N). Among the films prepared with banana peel extract, the sample BP_A prepared with only alginate showed the highest value of firmness (25.0 N), while in the case of the red beetroot peel extract, the sample RBP_RP, prepared with rice proteins, showed the highest resistance to local deformation (26.8 N). The tensile strength of edible films based on fruits and vegetables varies, with values between 0.03 and 30 MPa [38], and in the present study these values were at the lower limit. It is expected that a higher fruit and vegetable content will result in lower mechanical strength due to the plasticizing effect of short-chain sugars [38]. Sivarooban et al. [39] reported that the incorporation of grape seed extract in soy protein films significantly increased film puncture strength (from 2.5 to 5.3 N) and tensile strength (8.8–10.7 MPa) in comparison to the control film.

### 3.4. Bioactive Characterization of Edible Films

The results of the bioactive characterization of edible films, including determination of total phenolic content, antioxidant capacity, dopamine, and total betacyanin content, are presented in Table 4.

Among all formulated edible films, the most desirable bioactive profile, including TPC (BP: 17.40 mg GAE g^−1^ dw; RBP: 14.68 mg GAE g^−1^ dw) and antioxidant capacity (BP: 76.76 and 63.96 µmol TroloxE g^−1^ dw; RBP: 37.69 and 33.28 µmol TroloxE g^−1^ dw), was determined in the samples prepared with peanut proteins (RB_PEA and RBP_PEA). However, the highest dopamine content was determined in the control sample prepared only with the alginate (BP_A; 11.49 mg g^−1^ dw) followed by film with incorporated pumpkin proteins (BP_PUM; 10.72 mg g^−1^ dw), while the sample prepared with peanut proteins (RBP_PEA) was the richest in betacyanins (175.58 mg betanin g^−1^ dw), indicating good cross-linking of these ingredients.

**Figure 8:**
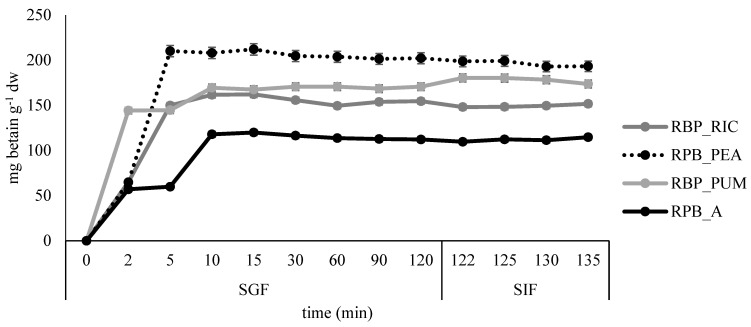
Release profile of betacyanins in simulated gastro-intestinal digestion for the edible films prepared from red beetroot peel extract.

**Figure 9:**
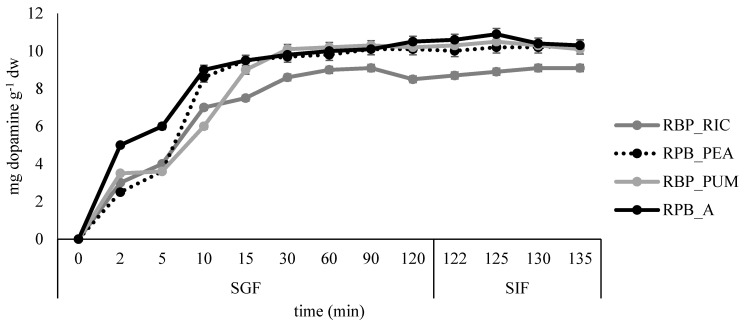
Release profile of dopamine in simulated gastro-intestinal digestion for the edible films prepared from banana peel extract.

All formulated films were subjected to simulated gastro-intestinal digestion. The results are presented in Figure 8 and Figure 9.

The fastest release of betacyanins was observed in sample RPB_PUM, where most of the betacyanins were released after 2 minutes in SGF, while in samples RBP_RIC and RPB_PEA, the release was prolonged to 5 minutes in SGF, and additionally to 10 min in the control sample RPB_A. After the highest release was reached, little or no additional release was observed during the remaining time in SGF and SIF. Similar results were reported by López de Lacey et al. [40], who noted a more rapid release of tea polyphenols from agar films at the beginning of digestion and a tendency to decrease significantly during gastric digestion, whereas little or no release was observed during intestinal digestion.

The slowest release of dopamine was observed in sample RPB_PUM where most of the dopamine was released after 15 min in SGF, while in all remaining samples the fastest dopamine release was observed during the first 10 min in SGF. The slight increase in dopamine release was continued during the remaining time in SGF and SIF for samples BP_A, BP_PEA, and BP_PUM, whereas for the sample BP_RIC distortion in release kinetics was observed at the end of SGF, but release increased slightly again during the time in SIF.

## 4. Conclusions

Banana and red beetroot peels were found to be natural raw materials with a high potential as a source of bioactive compounds (dopamine and betacyanins, respectively). Water extracts of banana and red beetroot peel did not show serious adverse effects on macromolecular models or CaCo2 and HepG2 human cells lines. Additionally, alginate in combination with plants proteins resulted in the formulation of edible films with satisfactory physical and mechanical properties, while extracts of banana and red beetroot peel showed great potential for wider applications in the food industry as a valuable source of bioactive compounds.

## Figures and Tables

**Table 1:** Formulations of edible films.

Plant Material	Sample Mark	Mass of Ingredients					
		Water (W) or Extract (E)	Alginate	Rice Proteins	Peanut Proteins	Pumpkin Proteins	Glycerol
Control	C_A	19.2 g W	0.8 g	/	/	/	0.2 g
	C_RP	19.2 g W	0.8 g	0.2 g	/	/	0.2 g
	C_PB	19.2 g W	0.8 g	/	0.2 g	/	0.2 g
	C_PP	19.2 g W	0.8 g	/	/	0.2 g	0.2 g
Banana peel	BP_A	19.2 g E	0.8 g	/	/	/	0.2 g
	BP_RP	19.2 g E	0.8 g	0.2 g	/	/	0.2 g
	BP_PB	19.2 g E	0.8 g	/	0.2 g	/	0.2 g
	BP_PP	19.2 g E	0.8 g	/	/	0.2 g	0.2 g
Red beetroot peel	RBP_A	19.2 g E	0.8 g	/	/	/	0.2 g
	RBP_RP	19.2 g E	0.8 g	0.2 g	/	/	0.2 g
	RBP_PB	19.2 g E	0.8 g	/	0.2 g	/	0.2 g
	RBP_PP	19.2 g E	0.8 g	/	/	0.2 g	0.2 g

**Table 2:** Bioactive characterization of banana and red beetroot peel extracts.

Sample	TPC (mg GAE L^−1^)	Antioxidant Capacity (mmol TroloxE L^−1^)		Dopamine (mg L^−1^)	Total Betacyanin Content (mg Betanin L^−1^)
		ABTS	DPPH		
Banana peel extract	507.1 ± 9.8	2.34 ± 0.02	1.52 ± 0.02	156.8 ± 1.1	/
Red beetroot peel extract	239.6 ± 5.0	1.69 ± 0.04	1.04 ± 0.01	/	90.1 ± 0.1

TPC-total phenolic content; GAE-gallic acid equivalents; TroloxE-trolox equivalents.

**Table 3:** Physical and mechanical parameters of formulated edible films.

Sample	Sample Mark	Soluble Dry Matter (%)	Thickness (µm)	L*	a*	b*	Δ*E*	Firmness (N)	Tensile Strength (MPa) *
Control	C_A	92.7 ± 0.9 ^ab^	110.7 ± 14.5 ^abcdefgh^	92.6 ± 0.4	−0.4 ± 0.0 ^abcdefg^	4.5 ± 0.3	/	30.8 ± 3.5 ^a^	0.15 ± 0.02
	C_RIC	75.0 ± 0.7 ^c^	105.3 ± 23.5 ^aijklmn^	88.0 ± 0.1 ^abc^	0.5 ± 0.0 ^ahijkl^	12.7 ± 0.5 ^abcdef^	9.5 ± 0.2	22.3 ± 0.3	0.11 ± 0.00
	C_PEA	86.1 ± 0.4 ^d^	153.3 ± 3.9 ^bopr^	88.2 ± 1.4 ^ade^	−0.3 ± 0.1 ^bhmnopr^	9.6 ± 0.4 ^aghis^	6.7 ± 0.5 ^a^	19.8 ± 1.8	0.10 ± 0.01
	C_PUM	90.8 ± 0.6 ^a^	56.7 ± 12.7 ^sštuv^	89.5 ± 0.1 ^bd^	−1.7 ± 0.0 ^cmsšt^	10.1 ± 0.2 ^bgjklš^	6.5 ± 0.3 ^a^	31.0 ± 1.1 ^a^	0.16 ± 0.00
Banana peel (BP)	BP_A	81.8 ± 0.9 ^e^	90.7 ± 10.6 ^ciszž123^	80.5 ± 0.4 ^f^	0.6 ± 0.1 ^dinuvz^	15.9 ± 0.3 ^mn^	16.6 ± 0 ^b^	25.0 ± 1.2 ^b^	0.13 ± 0.01
	BP_RIC	80.2 ± 1.0 ^e^	119.3 ± 33.5 ^djoz456^	84.0 ± 0.4 ^fg^	−0.2 ± 0.0 ^ejosuž1^	13.1 ± 0.5 ^cjmo^	12.1 ± 0.2	15.5 ± 0.4	0.08 ± 0.00
	BP_PEA	85.2 ± 0.6 ^d^	138.8 ± 15.9 ^ekp47^	81.0 ± 0.4	−1.0 ± 0.0 ^fkpšvž2^	15.1 ± 0.6 ^dno^	15.7 ± 0.2 ^b^	12.3 ± 0.5	0.06 ± 0.00
	BP_PUM	94.6 ± 0.4 ^bf^	63.7 ± 5.3 ^šž8β^	86.0 ± 1.0 ^ceg^	−0.5 ± 0.0 ^glrtz12^	9.6 ± 1.0 ^ehkpr^	8.3 ± 0.4	5.1 ± 0.1 ^c^	0.03 ± 0.00
Red beetroot peel (RBP)	RBP_A	74.1 ± 0.3 ^cg^	81.3 ± 5.5 ^flš159αδ^	52.4 ± 0.4	25.9 ± 0.5	9.6 ± 0.3 ^filpt^	48.2 ± 0.7 ^c^	5.4 ± 0.7 ^c^	0.30 ± 0.00
	RBP_RIC	67.0 ± 0.3	71.7 ± 4.3 ^gmu289δ^	58.9 ± 0.7	15.0 ± 0.4	20.4 ± 0.7	40.3 ± 0.1	26.8 ± 0.2 ^b^	0.13 ± 0.01
	RBP_PEA	75.1 ± 0.8 ^g^	124.2 ± 14.7 ^hnr367α^	61.9 ± 0.5	16.0 ± 0.2	26.7 ± 0.4	38.4 ± 0.3	3.0 ± 1.0	0.02 ± 0.00
	RBP_PUM	95.2 ± 0.9 ^f^	40.2 ± 5.1 ^vβγδ^	55.9 ± 1.8	28.9 ± 2.3	9.0 ± 1.5 ^rsšt^	47.2 ± 0.4 ^c^	7.5 ± 0.6	0.04 ± 0.01

Means denoted with the same superscript letter in the same column are not statistically significantly (*p* > 0.05) different; * no statistically significant difference (*p* > 0.05) between any of the samples was observed in the means of tensile strength.

**Table 4:** Bioactive characterization of edible films.

Sample	Sample Mark	TPC (mg g^−1^ dw)	Antioxidant Capacity (µmol TroloxE g^−1^ dw)		Dopamine (mg g^−1^ dw)	Total Betacyanin Content (mg Betanin g^−1^ dw)
			ABTS	DPPH		
Banana Peel (BP)	BP_A	13.00 ± 1.38 ^a^	38.28 ± 4.48	28.60 ± 0.45	11.49 ± 0.12 ^a^	/
	BP_RIC	13.27 ± 1.07 ^a^	44.19 ± 3.89	34.88 ± 1.34	9.77 ± 0.08 ^b^	/
	BP_PEA	17.40 ± 1.00 ^b^	76.76 ± 0.24	63.96 ± 2.55	10.46 ± 0.62 ^bc^	/
	BP_PUM	16.90 ± 0.46 ^b^	51.51 ± 8.95	43.42 ± 1.06	10.72 ± 0.17 ^ac^	/
Red Beetroot Peel (RBP)	RBP_A	8.52 ± 0.06 ^a^	24.84 ± 0.79 ^a^	16.30 ± 1.76 ^a^	/	101.04 ± 0.09
	RBP_RIC	9.13 ± 0.18 ^ab^	26.69 ± 2.85 ^a^	20.24 ± 0.22 ^ab^	/	128.05 ± 0.13
	RBP_PEA	14.68 ± 0.68	37.69 ± 0.75	33.28 ± 3.58	/	175.58 ± 1.39
	RBP_PUM	9.93 ± 0.67 ^b^	31.73 ± 1.12	23.67 ± 1.23 ^b^	/	134.20 ± 1.31

TPC-total phenolic content; GAE-gallic acid equivalents; TroloxE-trolox equivalents; dw-dry weight. Means denoted with the same superscript letter in the same column within the same sample (BP or RBP) are not statistically significantly (*p* > 0.05) different.

## Data Availability

The data presented in this study are available on request from the corresponding author.
